# Applying the Analytic Hierarchy Process in healthcare research: A systematic literature review and evaluation of reporting

**DOI:** 10.1186/s12911-015-0234-7

**Published:** 2015-12-24

**Authors:** Katharina Schmidt, Ines Aumann, Ines Hollander, Kathrin Damm, J.-Matthias Graf von der Schulenburg

**Affiliations:** Center for Health Economics Research Hannover (CHERH), Leibniz University of Hanover, Otto-Brenner-Str. 1, 30159 Hannover, Germany; Institute for Risk and Insurance, Leibniz University of Hanover, Otto-Brenner-Str. 1, 30159 Hannover, Germany

**Keywords:** Multi-criteria decision making, Priorities, Analytic Hierarchy Process, Methodological standards, Systematic literature review

## Abstract

**Background:**

The Analytic Hierarchy Process (AHP), developed by Saaty in the late 1970s, is one of the methods for multi-criteria decision making. The AHP disaggregates a complex decision problem into different hierarchical levels. The weight for each criterion and alternative are judged in pairwise comparisons and priorities are calculated by the Eigenvector method. The slowly increasing application of the AHP was the motivation for this study to explore the current state of its methodology in the healthcare context.

**Methods:**

A systematic literature review was conducted by searching the Pubmed and Web of Science databases for articles with the following keywords in their titles or abstracts: “Analytic Hierarchy Process,” “Analytical Hierarchy Process,” “multi-criteria decision analysis,” “multiple criteria decision,” “stated preference,” and “pairwise comparison.” In addition, we developed reporting criteria to indicate whether the authors reported important aspects and evaluated the resulting studies’ reporting.

**Results:**

The systematic review resulted in 121 articles. The number of studies applying AHP has increased since 2005. Most studies were from Asia (almost 30 %), followed by the US (25.6 %). On average, the studies used 19.64 criteria throughout their hierarchical levels. Furthermore, we restricted a detailed analysis to those articles published within the last 5 years (*n* = 69). The mean of participants in these studies were 109, whereas we identified major differences in how the surveys were conducted. The evaluation of reporting showed that the mean of reported elements was about 6.75 out of 10. Thus, 12 out of 69 studies reported less than half of the criteria.

**Conclusion:**

The AHP has been applied inconsistently in healthcare research. A minority of studies described all the relevant aspects. Thus, the statements in this review may be biased, as they are restricted to the information available in the papers. Hence, further research is required to discover who should be interviewed and how, how inconsistent answers should be dealt with, and how the outcome and stability of the results should be presented. In addition, we need new insights to determine which target group can best handle the challenges of the AHP.

## Background

The resources in health care systems are limited. Exacerbating this issue is the problem that many developed countries face, that is, the rising proportion of older, multimorbid patients, who serve to raise the cost of health care. Furthermore, innovations in medical care, such as equipment, pharmaceuticals, and treatment methods, are also driving up costs. German politicians have adopted new laws to manage the costs of pharmaceuticals, e.g. the Act on the Reform of the Market for Medicinal Products in 2011 (in German: AMNOG [1]). In this context, patient-relevant outcomes have drawn greater attention because the added benefit for patients determines the reimbursement price. But also, other countries are interested in reliable methods to measure benefits for patients, for example, to support Health Technology Assessments by patient preferences [2, 3]. Therefore, while it is now important to measure the benefits and to prioritize the needs of patients, it will be even more so in the future. However, several studies have found a divergence in patients’ and physicians’ preferences or priorities regarding prevention and therapy (e.g. [4–6]). Thus, one mean of evaluating these preferences and bringing them into accord is to take the required perspective for the situation. In order to find appropriate methods for measuring the benefits and for prioritizing them, beside the established methods, new approaches of decision making tools are transferred from other fields of research, like the marketing sector. For all of these methods it is essential to measure the trade-off between attributes in multi-criteria decision situations for each participant or the group, and as such, adequate and understandable methods are essential.

Several methods are known for multi-criteria decision making in the field of health care, including value based methods, strategy based methods, and conjoint analyses [7]. In Germany, the Institute for Quality and Efficiency in Health Care (IQWiG) suggested two methods for multi-attribute decision making: Conjoint Analysis (CA) and the Analytic Hierarchy Process (AHP) [8]. Although they concluded that both methods are applicable for decision making, they were also confronted with methodological limitations. As the advantages and disadvantages of established methods like the CA have been discussed in a number of publications (e.g. [9–11]), the AHP method has received less attention. Therefore, we wanted to figure out whether the AHP method could become a good alternative in multi-criteria decision making.

### Relevance and objective of the study

The Analytic Hierarchy Process (AHP) was developed by Saaty in the late 1970s and originally was applied to the marketing sector [12, 13]. Dolan et al. were the first to apply this method to health economics research in 1989 [14, 15]; since then, it has been accepted slowly as a method in the field of multi-criteria decision making in healthcare. Liberatore and Nydick described the importance of applying the AHP as follows: “Health care and medical decision making has been an early and on-going application area for the AHP” [16]. The AHP method was applied to different contexts, for example, the development of clinical guidelines [17, 18] or biomedical innovations and technology development [19, 20].

The increasing application of the AHP has been the motivation for this study to explore the current state of its methodology. The method is the basis for assessing the best instrument for each decision situation and reflecting each participant’s opinion correctly. A review provides an overview of published papers in this field. In line with De Bekker-Grob et al. [21], we provide a systematic review of the AHP. Therefore, an overview is given of the year of publication, country, and number of criteria used in the AHP (Section 3). In addition, Hummel and Ijzerman [22] analyzed the thematic field in which AHP is used. They identified the different areas of application (e.g., shared decision making, clinical guidelines, and healthcare management), number of criteria and alternatives, individual or group decisions, participants, and rating method. We focus on the methodological applications in the second step. In addition, the analyzed time horizon (2010–2015) should provide an update on Hummel and Ijzerman’s study and allow us to provide details of the most recent developments in the subject area. As in Mühlbacher’s overview [23], the field of application and the sample are inspected, although our focus remains on the current state of the research (the last 5 years) and the reporting of methodological aspects in the papers. In addition, the evaluation of studies’ reporting allows deeper insights. Therefore, we develop criteria for reporting the AHP method and determine to what extent the studies fulfill the criteria. We conclude by proposing recommended situations in which the AHP can be used.

### AHP – a short introduction

As a short introduction into the method of AHP, we report the most important aspects here. We refer to detailed papers to provide deeper insights into specific methodological aspects.

**Fig. 1:**
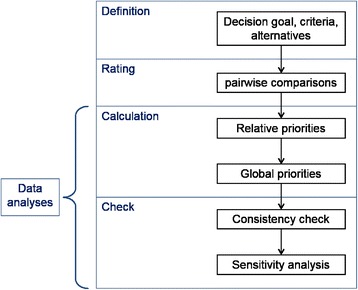
Steps of the AHP (modeled after Dolan et al. [25] and Dolan [7]])

The AHP disaggregates a complex decision problem into different hierarchical levels (see Saaty’s axioms for the AHP [24]). The application of an AHP is structured into six steps (see also Fig. 1), suggested by Dolan et al. [25] and Dolan [7], as follows: 1. define the decision goal, criteria, and alternatives, 2. rate the criteria in pairwise comparisons, 3. calculate the relative priority weights for the (sub-)criteria, 4. calculate the criteria’s global priority weights and combine the alternatives’ priorities, 5. control for inconsistency, and 6. perform sensitivity analysis.

At the first hierarchical level, the aim of the study is defined followed by the main criteria, which can be divided further at lower levels into sub-criteria. If necessary, alternatives that contain specific combinations of characteristics can be arranged at the lowest level of the hierarchy. Although the AHP was introduced for group decisions, it may also be applied to single person decisions [26]. Pairwise comparisons at each hierarchical level present the judgments and they must be evaluated according to a scale developed by Saaty, which ranges from 9 to 1 to 9. If the alternatives consisted of subjective combinations of the criteria, the alternatives would be judged also with regard to each criterion. Saaty provided a detailed description of his scale and its intensities [12].

In order to analyze the interviews, the pairwise comparisons of (sub-)criteria at each level are displayed in ordered schemes (matrixes). An example is seen in Saaty ([24], p. 164). Only half of the matrix has to be filled in, as the other half is obtained from the reciprocal weights. The Eigenvector method (EV) is the most common means of calculating the priority vector, although other methods, such as additive normalization, weighted least-squares, logarithmic least-squares, logarithmic goal programming, and fuzzy preference programming methods, yield comparable results [27]. The EV relies on the matrix’s principle eigenvalue, which results from a process of repeated squaring and normalization (for more information, see Srdjevic [27] or Saaty [12]). The resulting local weights describe the relative priorities in relation to their parent criterion. The local weights form the global weights for the criteria through multiplication with the local weights from their parent criteria [24]. Thereby, global weights for criteria show the importance of each criterion in the overall context of the hierarchy. The priorities for the alternatives of the AHP are calculated by the sum of the particular local and global weights for each alternative [23]. For detailed information and examples concerning the calculations, see Saaty [28].

The aggregation of the individual judgments or priorities is fundamental to the outcome of the study. The first option is to have the group of participants vote by finding consensus. Another alternative is to aggregate the individual judgments. Still further, the literature suggests finding the geometric mean [29] or arithmetic mean [30]. In addition, the timing of calculating the average affects the results [30], specifically, the average of participants’ judgments or the average of participants’ global weights. Yet another option is to give special weight to one participant’s decision on the basis of that participant being an expert in the field or holding an exceptional position within the group [30]. The consistency ratio (CR) measures the uniformity of a respondent’s answers to the AHP questions. Saaty [24] describes the calculation of the CR in detail. The CR can also be calculated for a group of respondents.

Although the AHP has been applied to a variety of topics within the healthcare field, the sensitivity analyses on hierarchical decision making has received little investigation [31]. It should be noted that there are two distinct types of sensitivity analysis, that of judgments and that of priorities [32]. The former has been explained and tested by Arbel [33], Moreno-Jimenez and Vargas [34], and Sugihara and Tanaka [35]. They determined the judgments’ upper and lower bounds and articulated the preferences through preference structures. Other approaches originate from Moreno-Jimenez and Vargas [34], Triantaphyllou and Sánchez [36], Sowlati et al. [37], Masuda [38], and Huang [39]. Erkut and Tarimcilar [40] provided “a collection of practical tools for a potentially powerful sensitivity analysis in the AHP”. In addition, Altuzarra et al. [41] proposed a method for determining the stability of group decisions. If the AHP includes alternatives, the sensitivity analysis could show the effect of varying weights on the alternatives’ rankings [23]. Therefore, potential rank reversal of alternatives can be simulated. Rank reversal occurs when adding or deleting an (irrelevant) alternative leads to a shift in the previous alternatives’ ranking order [42].

## Methods

This chapter is divided into two parts to introduce the methods used in this paper. The first part describes the method of the systematic review, which includes the key words and a flow chart. Further, in chapter 2.2, we describe our evaluation of reporting quality for the included studies.

### Systematic literature review

The basis of this review is a systematic literature research on the Pubmed and Web of Science databases (date of research: 10/27/2015). As we focused our research question on healthcare, we did not include further databases in the other scientific fields. We searched both databases for articles with the following keywords in their titles or abstracts: “Analytic Hierarchy Process,” “Analytical Hierarchy Process,” “multi-criteria decision analysis,” “multiple criteria decision,” “stated preference,” and “pairwise comparison.” We provided the search strategy in Appendix: Table 1. It was technically not possible to search Web of Science for keywords in the abstracts. We refined the search by including only articles written in German or English and those associated with healthcare. Two independent reviewers evaluated the titles and abstracts of the resulting studies. Therefore, the criterion for inclusion was that the article is the primary source and the study used the AHP method within the healthcare setting. Additionally, we conducted a manual search to find further articles not included in the aforementioned databases. Thereafter, the two reviewers screened the full texts of the remaining articles and discussed whether to include them in the review. After reaching consensus, the important information was summarized in a table (not shown). Apart from common information, like the author, title, publication year, country, and journal, we extracted additional information regarding the study’s aim, source of criteria identification, hierarchy design, form of implementation, and analytical steps in order to conduct our analysis. The results are described in Section 3 for the entire period and in detail for the last 5 years in Subsection 3.1. The first step should give a short overview of all studies that were conducted with AHP in health care. In the second step, we reported the current state of research in more detail.

### Evaluation of reporting quality

The papers identified from the last 5 years resulting from the systematic review were evaluated with regard to their reporting quality. Because there was no set standard by which to judge the AHP’s methodological issues, the evaluation of the studies’ quality was quite challenging. The before mentioned studies by De Bekker-Grob et al. [21], Hummel and Ijzerman [22], and Mühlbacher et al. [23] did not report quality criteria. However, the Consolidated Standards of Reporting Trials (CONSORT) Statement for randomized controlled trials [43] and the Preferred Reporting Items for Systematic Reviews and Meta-Analyses (PRISMA) Statement [44] may provide some direction by providing checklists for transparent and complete reporting. The reason why authors should report specific aspects is the traceability of the study. Some criteria from the CONSORT Statement could be transferred to AHP studies: sample size, participants (eligibility criteria), trial designs, and statistical methods. In the case of the AHP method, the latter criterion consists of the CR, the method used to calculate the weights, the statistical software, and sensitivity analyses. Another checklist item is the description of the intervention. Transferred to the AHP method, authors should provide information about the interview process. Besides, another guideline for good research practices is published by Bridges et al. [9]. They provide a detailed checklist that is specific for conducting conjoint analyses. Since it suggests quality aspects only for those kinds of studies, the checklist cannot be used directly for our evaluation. However, we summarized the recommendations from the different fields and we obtained a simplified measurement of reporting by counting the elements that were included in the studies. Therefore, we evaluated whether the authors mentioned aspects for the following elements in their papers:

- Decision goal, criteria (and if necessary alternatives)
- Number of participants
- Type of participants (patients, potential consumers, or experts)
- Decision making by group or individually
- Scale for pairwise comparisons
- Interview process (face to face, email, questionnaire, judgments based on literature)
- Software
- CR
- Calculation of weights
- Sensitivity analysis

The last criterion was valid only for studies including alternatives. Thus, for the other papers without alternatives, we could determine only whether descriptive statistics (e.g., standard deviation, SD and confidence intervals, CI) were reported for the judgments or weights. We calculated the sum of all reported aspects for each study and present the results in Appendix: Table 2 and we show charts in Subsection 3.2. Nevertheless, we could not evaluate the content of each of the abovementioned criteria but only whether the criteria were reported in the study.

## Results

**Fig. 2:**
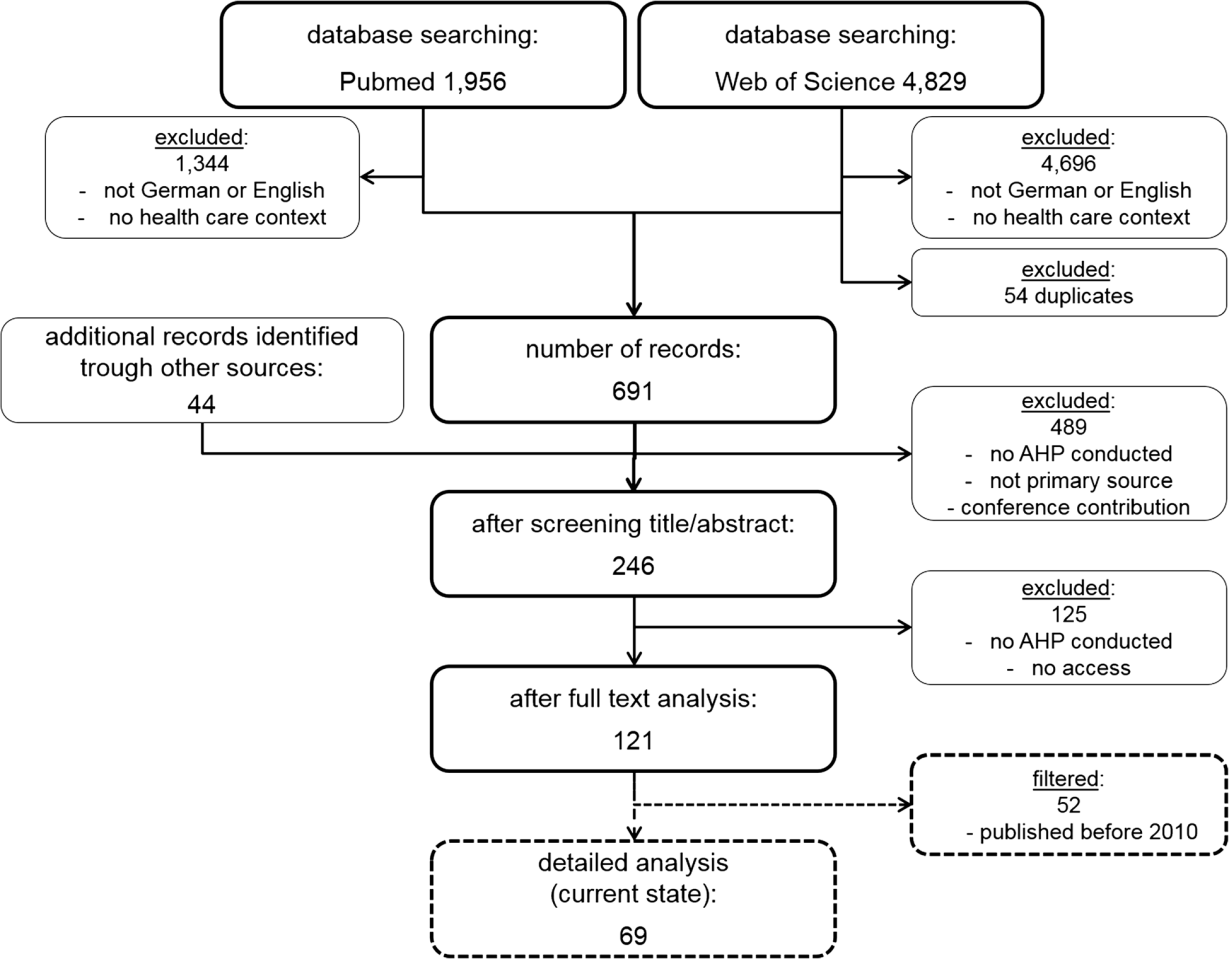
Flow Chart of the Systematic Literature Review

The search in Pubmed yielded to 1,956 articles and the search in Web of Science yielded to 4,829 articles, as Fig. 2 shows. Furthermore, 44 additional records were found via manual search. By screening titles and abstracts, we limited the sample to 246 articles (we excluded a total of 6,485 articles based on language or irrelevance to healthcare and we found 54 duplicates). Thereafter, we examined the full articles in order to determine whether they apply AHP to the field of healthcare. An additional 125 papers were excluded because they were not original studies or they used other stated preference methods (e.g., discrete choice experiment). In total, this process yielded to 121 relevant studies; the Appendix: Table 3 provides a complete list. We provide a brief overview of these studies to show how many studies have been published in this field and in which context the authors used the AHP. In addition, the overview presents the development and the relevance to the AHP method. In order to explore the current state of the literature, we limited the body of our research to articles published within the last 5 years. This restriction reduced the number of studies to 69. The detailed analysis of these studies’ methodologies made it necessary to reduce the number of articles.

For a first overview, we briefly summarized the key factors of all of the relevant articles (*n* = 121), such as their publication year, country, number of attributes, and levels.

**Fig. 3:**
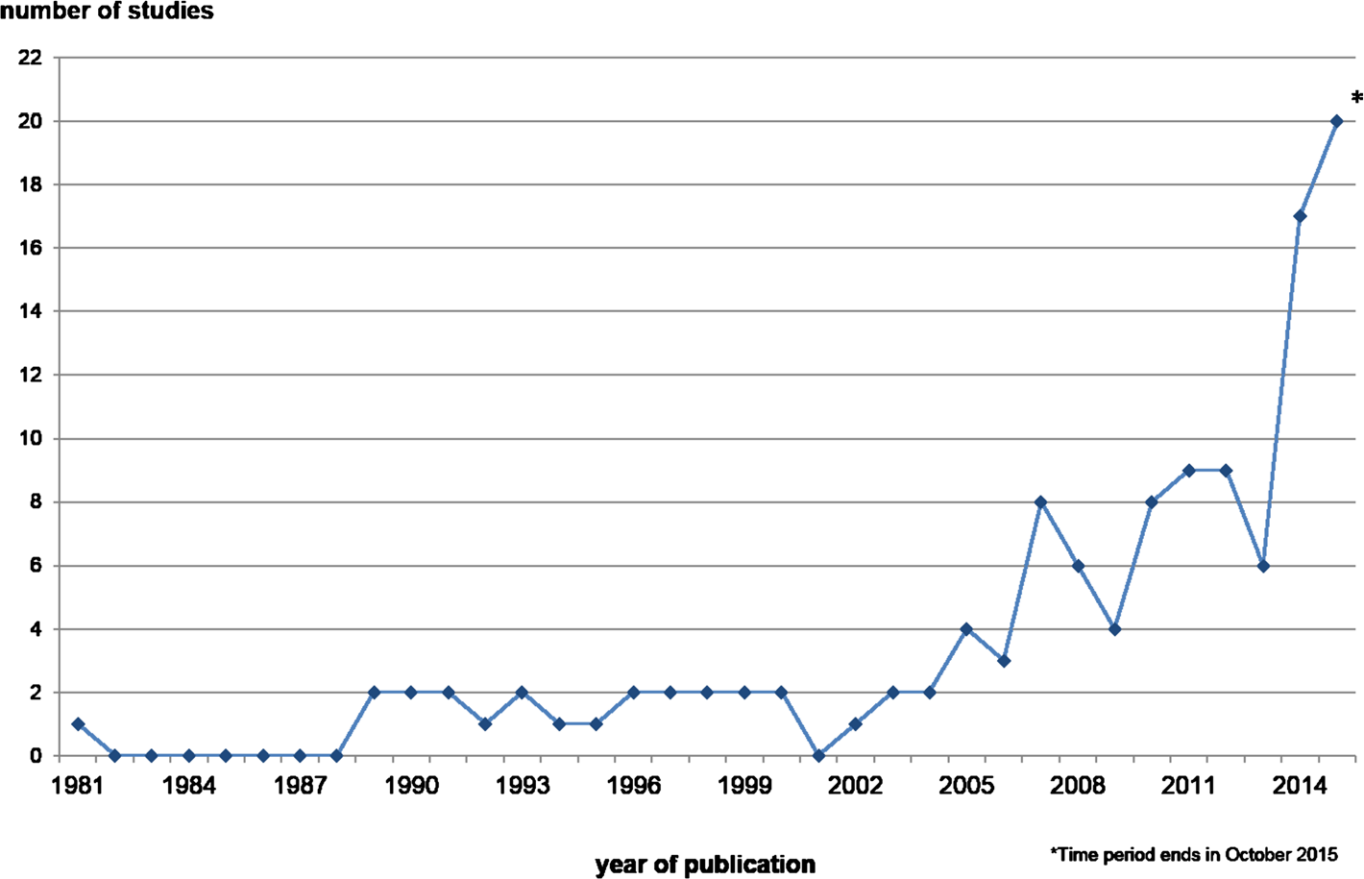
Included Studies by Year of Publication

The earliest study to use the AHP was published in 1981, but the AHP has become increasingly popular since 2005 (see also Fig. 3). The 2 years with the greatest number of studies published on the subject were 2011 and 2012 with nine each. However, it should be noted that our evaluation period contains only the first 10 months of 2015, in which as many as 20 studies were published. On average, there were 2.5 studies per year between 1981 and 2013. During the 1990s, there was an average of 1.7 publications on the AHP per year, which increased to 4.6 per year between 2000 and 2013. In 2014 and 2015 the average increased to the peak of 18.5 studies, although the last two months of 2015 are not included.

Most studies were from Asia (29.75 %), followed by the US (25.62 %). Almost all studies published before 2000 were conducted in the US (*n* = 15). However, between 2000 and 2010, a larger proportion came from Asia (*n* = 8) and Europe (*n* = 7), although most were still from the US (*n* = 8). Since 2010, Asia (*n* = 26) and Europe (*n* = 17) have surpassed the number of publications in the US (*n* = 8).

**Fig. 4:**
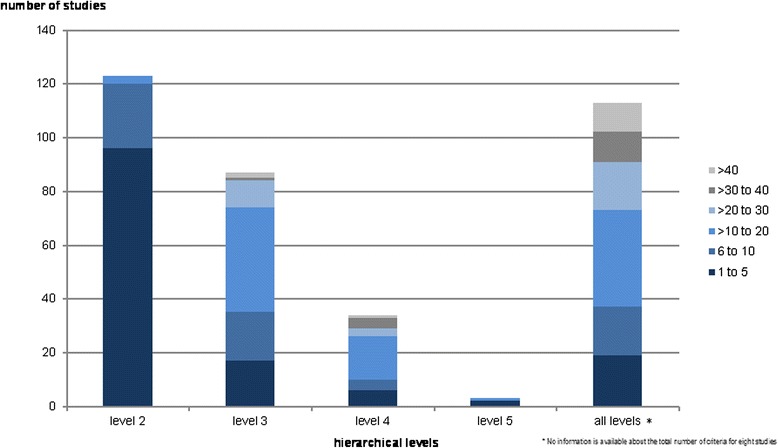
Number of Criteria per Hierarchical Level

Another important aspect of these studies is the number of hierarchical levels that they include. Therefore, the studies could include more than one hierarchy, so in some cases the number of studies did not sum up to 121. More than half of the studies (51 %) included three hierarchical levels, 23 % described their hierarchy with two levels, and 21 % used four levels. On average, the studies used 19.76 criteria throughout their hierarchal levels. At the second hierarchical level, 96 articles (78 %) included between 1 and 5 criteria (Fig. 4). At the third and fourth levels, most studies (*n* = 39 and *n* = 16 or 45 and 47 %, respectively) used between 11 and 20 criteria. The number of studies with five hierarchical levels was quite small (*n* = 3). As expected, the number of criteria increases as the hierarchical level increases. The right bar in Fig. 4 shows the total number of criteria for all hierarchical levels per study.

Following the method set forth by Hummel and Ijzerman [22], we divided the studies into five categories: development of clinical guidelines, healthcare management, government policy, shared decision making, and biomedical innovation. We classified 38 studies (31 %) as pertaining to the development of clinical guidelines or recommendations, 30 (25 %) to healthcare management, 26 (21 %) to government policy, 15 (12 %) to biomedical innovation, and12 (10 %) to shared decision making.

### Detailed analysis of the current state of research

This subsection summarizes the results of our analyses of the articles published within the last 5 years (January 2010 to October 2015). We examine how the studies design their hierarchies and carry out their surveys, and which analytical steps they take. In doing so, we follow the steps for conducting an AHP shown in Fig. 1.

#### Definition of decision goal, criteria, and alternatives

The first step in conducting an AHP is to define the decision goal and criteria that describe the goal at a lower hierarchical level. In order to do this, many studies relied on literature research [20, 25, 26, 45–83]. In addition, many studies relied on expert interviews [20, 45–49, 51, 54, 56–58, 61, 66–71, 74, 75, 77, 78, 81–97] or focus groups [26, 51, 69, 82, 87, 98]. Almost all of the studies defined their criteria by analyzing more than one source of information, although five publications did not explain their process for this step [99–103]. Some authors defined the criteria according to standards or established guidelines [25, 50, 52, 59, 80, 84, 92, 93, 104–108] or even from previous study results [25, 47, 62, 68, 69, 71, 72, 81]. Still other authors relied on their own expertise [64, 73, 107, 109, 110].

#### Judgment through pairwise comparisons

The sample sizes varied between one author who judged the AHP for himself [73, 107–109] to 1,283 participants [55]. In total, 50 of the 69 articles reported the number of participants in their AHP studies. The mean number of participants in these studies was about 109. Depending on the studies’ goal, the participants belonged to the following groups: hospital employees [49, 92], patients [25, 47, 55, 59, 60, 64, 69, 72, 75, 82, 95, 98], public/consumers [52, 70, 103], doctors or specialists [26, 71, 72, 74, 79, 81, 83, 93, 94, 96, 97, 99, 110], medical students [80] or teachers [77], biomedical engineers [94], technical experts [93], managers [93], administrators [20], and stakeholders [75]. Of the studies, 44 interviewed experts [20, 26, 45, 46, 48–51, 54, 56–58, 61, 62, 66–68, 71, 74, 76–79, 81, 83–94, 96, 97, 99, 104–107, 110], 11 studies surveyed consumers or patients [25, 47, 52, 55, 59, 60, 69, 70, 82, 98, 103], and four studies included both [64, 72, 75, 95]. However, six authors did not mention who answered the AHP questions [53, 63, 65, 100–102].

Next, we considered whether the AHP was applied at individual or group level. Most of the studies questioned their participants individually [20, 25, 26, 47, 55, 56, 59, 61, 62, 64, 66, 69–71, 74, 75, 77, 79–83, 87–90, 94, 97–99, 103, 104, 109–111]. On the other hand, only six articles mentioned group decisions [46, 49, 72, 84, 92, 96]. Five studies conducted individual judgments as well as group decisions [51, 60, 86, 93, 95]. The remaining 23 articles did not describe the judgment, or they had only one person who answered.

In addition, there were differences in the applied scales for the pairwise comparisons. As explained in Subsection 1.1, the original scale implemented by Saaty ranges from nine (or 1/9) to one to nine. This scale was adopted by 37 of the articles in our sample [25, 45, 46, 50–52, 54–57, 60–62, 66, 71–73, 75, 79, 80, 83, 84, 86–89, 91, 92, 94, 95, 97, 98, 102, 103, 107–109, 111]. Other studies used ranges between 1 and 4 [20, 59], 1 and 5 [67, 70, 106], 5 and 1 and 5 [26, 81, 90, 110], 6 and 1 and 6 [99],1 and 7 [47],1 and 9 [58, 77, 96], and 1 and 11 [74]. The remainder of the studies did not provide information about their scale [48, 49, 53, 63–65, 68, 69, 76, 78, 82, 85, 93, 104].

Furthermore, there were major differences in how the surveys were conducted. Once again, not all of the authors discussed their process in detail, but those that did so used online questionnaires [20, 47, 51, 55, 58, 70, 74, 75, 81–83, 111] (emailed) questionnaires [26, 59, 64, 66, 71, 77, 79, 80, 86, 91, 94, 95, 104, 110], face-to-face interviews [25, 45, 87, 90, 98], group discussions or workshops [49, 60, 64, 72, 84, 86, 92, 93, 96], or Delphi panel method [61].

#### Analysis and validation of results

Specific software can support the AHP design and further analyses. However, only 35 of the 69 studies (49.28 %) mentioned which software they used. The majority of the studies that reported software chose Expert Choice® (23.19 %), while others used such packages as Microsoft Excel [25, 77, 88, 90], or IBM SPSS Statistics [45, 53, 80, 99, 104]. In the last 5 years, a more diverse range of software packages has been in use; in addition to the aforementioned packages, researchers have chosen Super Decisions TM or Crystal Xcelsius [73, 107], or programmed their own software [20].

The detailed analysis showed that 22 out of the 69 studies did not state a CR. However, 31 studies used a CR of 0.1 [20, 26, 45, 46, 49–51, 56, 57, 60–62, 67, 71–74, 76, 77, 83, 87, 89, 91, 98–102, 107–109], five studies widened the range to a CR of 0.15 [25, 59, 64, 75, 111], and three studies accepted a CR of 0.2 or less [70, 81, 97]. The remaining studies did not establish a threshold prior to measuring average CRs [55, 80]. As a consequence of these consistency conditions, 14 of the studies reported the number of participants that must be excluded in order to meet their established threshold [47, 55, 59, 61, 63, 70–72, 75, 78, 81, 98, 99, 104]. However, only a small proportion of the studies actually outlined a procedure for dealing with excessive inconsistency (i.e., a CR above the established threshold). Chen et al. [70] and Pecchia et al. [26] asked the participants to fill out their questionnaires again. Hummel et al. [94], Suner et al. [83], Velmurugan et al. [102], and Cancela et al. [51] asked the participants to check and revise their decisions. Chung et al. [71], Li et al. [77], and Pecchia et al. [81] excluded the inconsistent participants from their analyses. Hou et al. [67] wrote that, in this case, “the judgment matrix has to be modified and recalculated.” Page et al. [80] ran simulations in which they assumed that the inconsistent answers were, in fact, consistent in the first place.

Furthermore, we examined group decision making. Danner et al. [72], Lin et al. [91], Papadopoulos et al. [56], Reddy et al. [86], Shojaei et al. [87], Jaberidoost et al. [66], and Hsu et al. [90] explored this topic by taking the geometric mean of the individual weights. Hilgerink et al. [93] and Hummel et al. [94] summarized the individual judgments with geometric means, and then, calculated the group weights. Conversely, other studies only averaged the group judgments [75, 95]. Olivieri et al. [79] presented two AHPs; in the first, they calculated geometric means for the ranks and in the second, they calculated the inter-participant, standardized, geometric means of the weights as well as the inter-participant means. Perseghin et al. [96], Uzoka et al. [97], and Kuruoglu et al. [98] aggregated the participants’ judgments according to the median, and then, calculated the weights. By contrast, Taghipour et al. [49] constructed the group judgments by using weighted means. Unfortunately, 40 of the studies did not describe their weight calculations in detail [20, 45–48, 50–55, 57, 58, 61–65, 67–70, 73, 74, 77–79, 82, 85, 88, 89, 96, 99–101, 103, 104, 106, 107, 110]. However, 39 authors mentioned that they used the EV [25, 26, 45–47, 49, 50, 55–57, 59, 60, 62, 65, 66, 71, 72, 75, 76, 80, 81, 83, 86–95, 97, 100, 102, 104, 105, 108, 109].

Very few of the studies (*n* = 14) examined the robustness of the weights [46, 53, 56, 73, 76, 78, 80, 82, 86, 93, 100, 101, 105, 107]. Diaz-Ledezma et al. [107] and Diaz-Ledezma and Parvizi [73] referred to Erkut and Tarimcilar [40], who introduced sensitivity analysis for the AHP. Hilgerink et al. [93] factored in uncertainty regarding the included criteria by asking participants to rate the sensitivity and specificity of the pairwise judgments on a three-point scale; this yielded negative, average, and positive scenarios for the overall priorities. The other studies did not mention efforts to account for uncertainty. Further studies conducted their sensitivity analyses with the graphics provided in Expert Choice ® [100, 101].

This subsection presents the most relevant aspects of conducting AHP, and thereby, reveals a high proportion of missing information from the literature. However, we summarize these facts in Subsection 3.2 and evaluate the number of reported aspects.

### Evaluation of reporting

In a final step, we evaluated the reporting of the studies (see Subsection 2.2). Therefore, we suggested ten criteria that the authors should address in their articles. Most of the aspects are described in Subsection 3.1, and so, we focus on the number of reported elements for evaluating the studies in this section. We evaluated the studies published between 2010 and 2015 (until the 27th of October) and the detailed table can be found in Appendix: Table 1. In addition, we summarized the most important aspects from the table in the following graphs.

**Fig. 5:**
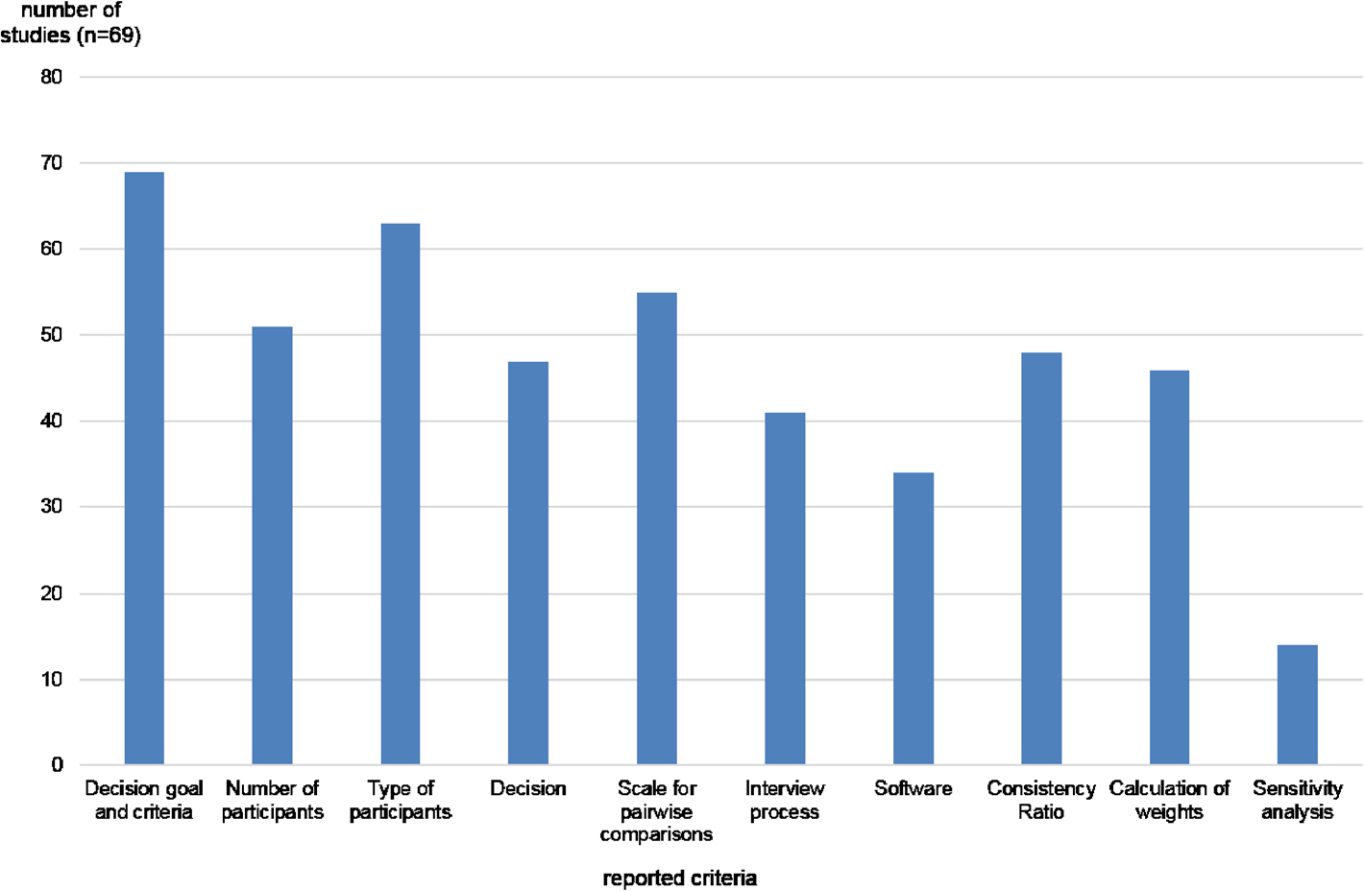
Number of Studies by the Reported Criteria

Figure 5 shows that all of the studies (*n* = 69) reported their decision goal and their criteria in their publications. However, several studies did not describe their interview process and did not mention which software they used. Particularly, only 15 out of 69 studies reported that they conducted sensitivity analysis.

The minimum number of reported criteria is one, namely, the study of Hsu et al. [63]. They described the aim of the study (assessment of oral phosphodiesterase type 5 inhibitors for treatment decisions of erectile dysfunction) and the hierarchy for the AHP but said nothing about the methods or study process. The studies that reported the highest number of ten criteria were published by Page [80] and Maruthur et al. [111]. The mean of the reported elements is 6.75, whereas only 12 out of 69 studies (17.39 %) reported less than half of the criteria.

**Fig. 6:**
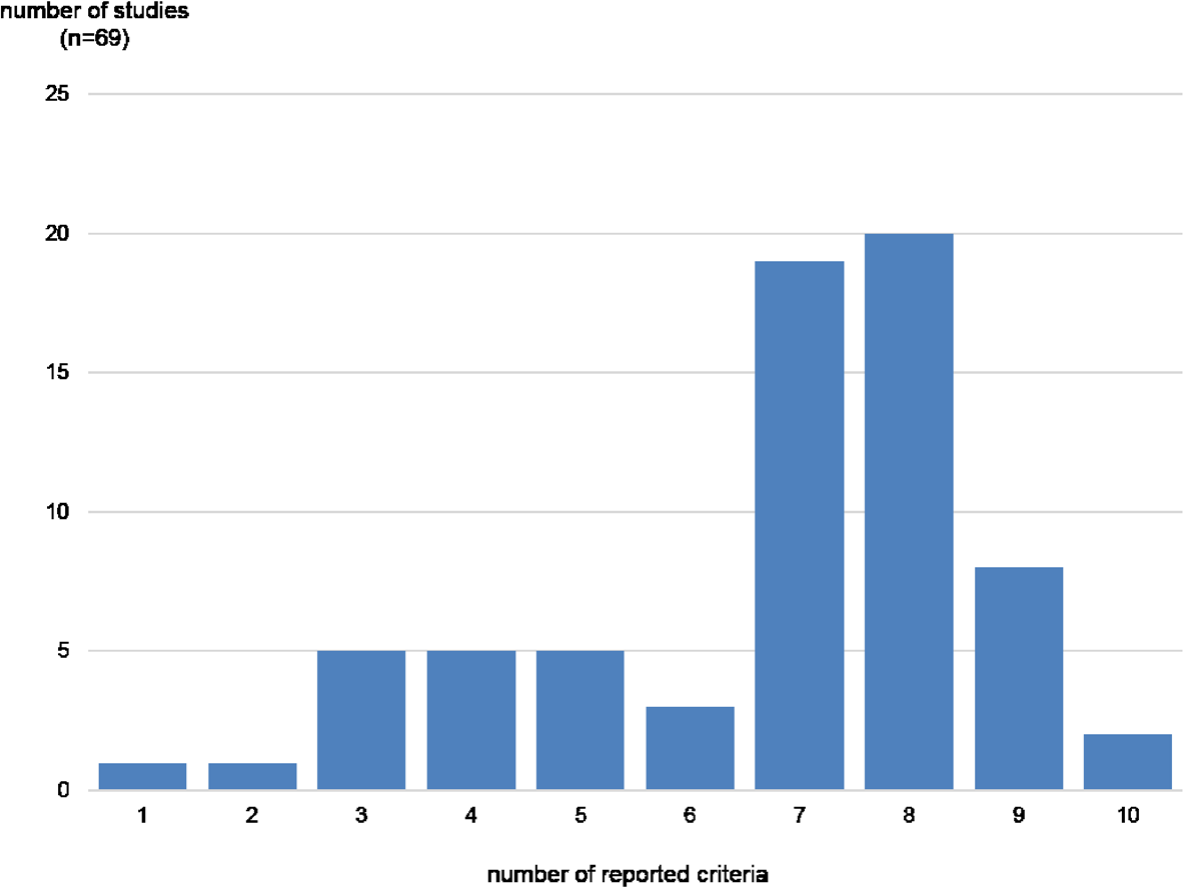
Evaluation Results for Reporting Quality

The next figure demonstrates the results from our evaluation of reporting quality (Fig. 6). This figure shows the results from our evaluation regarding the reporting quality of all publications between 2010 and 2015. The highest number of studies reached seven or eight points in the evaluation. Only a small number of studies (*n* = 2) reported one or two aspects required. However, two publications also reported all of the criteria. The mean of reported criteria is 6.75.

Furthermore, we divided the publications into two time periods because we wanted to examine whether the reporting quality has changed (not shown graphically). Therefore, we took the studies published between 2010 and 2013 and compared them with the recent state of research since 2014 (the peak of published studies seen in Fig. 3). In the last 2 years, five studies got nine points in comparison to only three studies in the early time period. Indeed, two publications from the last 2 years only reached one or two points compared to no publications between 2010 and 2013. As the mean of the reported criteria is 6.88 for the early period and 6.65 for the last 2 years. Apparently we do not see the expected increase of reporting quality.

## Discussion

As seen from the review, in the last 10 years (and particularly in the last 2 years), there has been a clear upward trend in the number of publications that apply the AHP to healthcare. One reason for this could be the increasing acceptance and the discussion about integration of this method into policy decision processes. For example, the IQWiG in Germany suggests the AHP in decision making regarding reimbursement as one appropriate method [8]. Currently, the development of clinical guidelines is the most popular subject for AHP studies, followed by healthcare management decisions.

In the first step, the authors have to decompose their research question and set up a hierarchy for the AHP. Therefore, we have seen that most of the authors rely on literature research and expert opinions. This proceeding could carry the risk to not including further important criteria that have not been covered before but that are important for the overall problem and for the complete hierarchy. In particular, the perspective of the participants (in contrast to previous research) could require new criteria for the AHP.

The review showed wide fields for choosing participants in the AHP studies, even though a large portion of papers described their samples as experts or potential consumers of goods or services in question. Sample size was an important factor in these studies, for while there is no precise rule, there is general consensus that the AHP does not require a particularly large sample [23]. Consequently, it should be noted that the results are not necessarily representative. The number of participants ranged from 1 (a single author who judged the AHP for himself) to almost 1,300 with the mean being about 109. This wide range could influence the studies’ results. The evaluation of reporting in Subsection 3.2 examined satisfactory reporting of the participants in most of the papers. However, common rules for the process should be developed and several of its aspects improved upon. For instance, future research should develop a standardized method for calculating the sample size. Furthermore, the identification of the correct study sample is imperative in order to answer the studies’ research question properly.

In some cases, the participants were invited to revise their answers in case of inconsistency, and thereby, participants could be unsettled and biased. However, inconsistent judging could also be an indicator of overstraining the participants. Furthermore, most of these studies carried out the AHP on an individual basis, whereas only four authors mentioned group decisions. This was an unexpected finding because the AHP was introduced initially to study group decisions. However, our evaluation of the studies’ reporting showed that only six authors did not mention whether they had conducted group or individual decisions. Moreover, the aggregation of the AHP results from the individual level to a group did not present a uniform set of results. The advantage of group consensus is that it allows for the discussion of pairwise comparisons, which, in turn, improves participants’ understanding of the problem and criteria, and thereby, participants answer less inconsistently. This is because, on the one hand, they discuss their decisions before they set their judgments, but on the other hand, it may be because of the consensus or average extreme judgments being compensated by the group. Thus, the quality of the decision, seen as consistency, is improved [112]. Otherwise, the composition of the group would be a highly influential factor in the process of reaching consensus. This is because individuals within the group could have opposite priorities or else could be unwilling to discuss their positions. In this case, it would not be possible to reach a unanimous vote. Thus, another alternative is to aggregate the individual judgments [113]. In order to do this, one may take the geometric mean or median of either the individual judgments or the individual weights. One prerequisite is that the reciprocal of the aggregated values must correspond to the individual reciprocal values [28]; this can be achieved only by taking the geometric mean [113]. Unfortunately, only 29 of the 69 studies describe their exact processes for calculating the weights, but 39 reported using the EV in some way.

Recently, researchers have paid some attention to whether the results of these studies are robust. Despite the fact that sensitivity analyses could offer more information on the problem of rank reversal as well as the interpretation of the outcome [23], only 14 out of the 69 studies that we examine reported conducting such tests [73, 76, 78, 82, 93, 107]. However, sensitivity analysis for AHP is relevant only when alternatives are included in the hierarchy. Consequently, 25 of 37 studies from our analysis missed reporting sensitivity analyses, as shown in Appendix: Table 2. One study without alternatives in the hierarchy suggested the use of standard deviations for weights [80]. The other sensitivity analysis presented in Subsection 1.1 requires a firm understanding of matrix algebra, does not yield fast or easy solutions, and is not supported by any software package. Although Expert Choice® provides the opportunity for sensitivity analysis, it offers only graphical simulation of one weight at the first hierarchical level [31]. Despite these challenges, sensitivity analyses remain vitally important as they allow researchers to assess the robustness of judgments, identify critical criteria or alternatives, find consensus through a range of judgments, and investigate different scenarios that support the decision [31]. Recently, Broekhuizen et al. have taken a further step concerning sensitivity analysis by providing an overview of dealing with uncertainty in multi-criteria decision making [114]. The results from sensitivity analysis can indicate potential rank reversal. The long-running dispute of rank reversal in AHP raised the question of “[…] the validity of AHP and the legitimacy of rank reversal” [42]. Wang et al. [42] argued that rank reversal is not only a phenomenon in the AHP but also in other decision making approaches. Saaty stated that the relative measurement of alternatives in the AHP implied by definition that all included alternatives were relevant, in contrast to utility theory that could face rank reversal problems [115]. Apart from these fundamental questions, several authors have suggested modifications to the AHP to overcome the problem of rank reversal [116].

Our evaluation of the reported criteria emphasizes the need to increase the number of given information in AHP studies. In general, authors should improve reporting on methodology, which is essential for comprehending and reproducing other authors’ results. This would serve to facilitate other researchers’ evaluations of study quality. In our opinion, two central explanations are possible for the current underreporting in the literature. First, the AHP, being fairly new, has few precisely formulated methodological rules. Second, what rules there are do not hold in practice. The latter observation also encompasses cases in which the AHP was too difficult for participants, either because of the formulations of the criteria or because of the method itself. It can be concluded that further research, in particular, methodological research, is needed in this field.

Although this study is based on systematic literature research and transparent evaluation criteria, there are a number of limitations that bear mentioning. As we primarily conducted our research on the Pubmed and Web of Science databases, it is possible that we did not include all relevant articles from other databases, even though we conducted a manual research. In addition, not all studies reported their procedures and methodologies in detail; therefore, the resulting statements in this review and the evaluation of the studies’ reporting could be biased, as we were restricted to available information. We are unable to make statements about the appropriateness of the evaluated content, like the sample size. By contrast, our evaluation criteria considered only whether a point was mentioned. Furthermore, the evaluation of reporting relied on the CONSORT and PRISMA Statements in order to develop criteria for the AHP. These statements suggest evaluation criteria for RCTs and systematic literature reviews, thus it could be criticized that we apply them to the subjective method of the AHP. The importance of each criterion can be criticized and our overall evaluation provides only an indication of the studies’ reporting with respect to informational content—not the quality. Moreover, we summarized the articles’ procedures but were unable to convey their results without some adaptions and generalizations; some aspects of the AHP must be adapted to suit the situation.

## Conclusion

We found that there is a pressing need to develop methodological standards for the AHP; otherwise, discrepancies in methodology could bias studies’ results. In particular, future research should establish a standard procedure for aggregating individual data, specifically, a standard for using the geometric mean versus the arithmetic mean and aggregating judgments or priorities. We should place special emphasis on finding practical sensitivity analysis to address the criticisms regarding rank reversal due to changed judgments. In addition, suggestions are necessary for reporting the robustness of weights for AHPs that do not include alternatives.

Besides the methodological aspects of the AHP, we should also think about the topic that is researched. We carved out that the AHP is based on the hierarchical structure and the criteria that are included. If the author uses improper assumptions, he will find biased results. Therefore, the AHP hierarchy should not only base on one source of information but also on a combination of different methods (e.g. literature research and expert interview). Hence, further research is required about how to determine the interviewees, what should be done with inconsistent answers, and how the outcomes and the stability of the results should be presented. In the future, we need new insights as to which target groups can best handle the challenges of the AHP. These challenges are mainly consistent answering, preventing overstraining by using adequate numbers of pairwise comparisons, and deciding between group and individual AHP. Therefore, researchers should investigate specific groups, like elderly people, healthy people, and patients with different diseases or disabilities.

In our study, we analyzed whether authors reported important aspects of the AHP in their studies. This could be a first step to evaluate the quality of studies applying AHP in healthcare. In addition, guidelines should be formulated as to which statistics should be reported and how to conduct high-quality AHPs. As mentioned before, Bridges et al. published a checklist that contains recommendations for conducting conjoint analyses on healthcare topics on behalf of the International Society for Pharmacoeconomics and Outcomes Research (ISPOR) group [9]. Besides aspects for study presentation, it suggests criteria for evaluating the choice of attributes and the appropriateness of the method for the research question. Still further, we should take the current criticisms of the AHP into consideration so that we can find solutions to address them.

This systematic literature review shows a heterogeneous picture for application of the AHP in health economics research. It is likely that interest in the AHP will rise in the future, particularly in its application to health economic evaluations, the weighing of therapy outcomes, and benefit assessments. In this context, the AHP method could support decision making regarding reimbursement of pharmaceuticals. This is largely owing to its ability to translate complex questions into stepwise comparisons at different hierarchical levels. In these hierarchies, both quantitative and qualitative criteria can be compared, which provides a more accurate representation of real-world healthcare issues. Therefore, it should be used for complex decision problems that can completely be decomposed into a hierarchical structure. Thus, patients could apply the AHP to clarify their priorities. The patients could also benefit from these structured decisions in conversations with their physicians. The second important point is to figure out by researches which are the appropriate participants that are able to judge this research problem reliably.

## Appendix

**Table 1:** Key words for systematic literature review

	Search terms	Pubmed	Web of Science
Block A	Analytic Hierarchy Process	481	10,127
	Analytical Hierarchy Process	486	3,148
	multi-criteria decision analysis	236	2,821
	multiple criteria decision	2,135	8,291
	stated preference	977	32,773
	Expert Choice	2,676	5,601
	pairwise comparison	2,873	10,385
Block B	Health economics	283,801	10,684
	Health care	1,346,972	412,669
Combination Block A	Analytic Hierarchy Process OR Analytical Hierarchy Process OR multi-criteria decision analysis OR multiple criteria decision OR stated preference OR Expert Choice OR pairwise comparison	9,685	68,767
	(Analytic Hierarchy Process[Title/Abstract]) OR (Analytical Hierarchy Process[Title/Abstract]) OR (multi-criteria decision analysis[Title/Abstract]) OR (multiple criteria decision[Title/Abstract]) OR (stated preference[Title/Abstract]) OR (Expert Choice[Title/Abstract]) OR (pairwise comparison[Title/Abstract] )	1,966	4,923
	(Analytic Hierarchy Process[Title/Abstract]) OR (Analytical Hierarchy Process[Title/Abstract]) OR (multi-criteria decision analysis[Title/Abstract]) OR (multiple criteria decision[Title/Abstract]) OR (stated preference[Title/Abstract]) OR (pairwise comparison[Title/Abstract] )	1,956	4,829
Block A AND Block B	(Analytic Hierarchy Process OR Analytical Hierarchy Process OR multi-criteria decision analysis OR multiple criteria decision OR stated preference OR pairwise comparison) AND health care	306	137
	((Analytic Hierarchy Process[Title/Abstract]) OR (Analytical Hierarchy Process[Title/Abstract]) OR (multi-criteria decision analysis[Title/Abstract]) OR (multiple criteria decision[Title/Abstract]) OR (stated preference[Title/Abstract]) OR (Expert Choice[Title/Abstract]) OR (pairwise comparison[Title/Abstract])) AND health care	307	139
Final search	(Analytic Hierarchy Process[Title/Abstract]) OR (Analytical Hierarchy Process[Title/Abstract]) OR (multi-criteria decision analysis[Title/Abstract]) OR (multiple criteria decision[Title/Abstract]) OR (stated preference[Title/Abstract]) OR (pairwise comparison[Title/Abstract]) Filter: Language English, German	1,839	4,474

**Table 2:** Evaluation of reporting quality

Authors	Year	Decision goal, criteria (and alternatives)	Number of participants	Type of participants	Decision	Scale for pairwise comparisons	Interview process	Software	CR	Calculation of weights	Sensitivity analysis	Reported elements
Ajami S, Ketabi S [92]	2012	yes	3 hospitals	E	g	9–1–9	f2f	Expert Choice®	n/a	EV, GA	n/a (alt)	8
Bahadori M et al. [117]	2014	yes	48	E	g	9–1–9	nominal group technique	Expert Choice®	1	n/a	n/a (alt)	8
Basoglu N et al. [69]	2012	yes	14	P	ind	n/a	n/a	n/a	n/a	n/a	n/a (alt)	4
Bi Y, Lai D, Yan H [45]	2010	yes	n/a	E	n/a	1–9	f2f	SPSS	0.1	EV	n/a	6
Cabrera-Barona P et al. [50]	2015	yes	32	E	n/a	9–1–9	n/a	n/a	0.1	n/a	n/a	5
Cancela J, Fico G, Arredondo Waldmeyer MT [51]	2015	yes	16	E	ind + g	1–9	online	BPMSG	0.1	n/a, median	n/a	9
Chen L et al. [70]	2014	yes	102	C	ind	1–5	online	n/a	0.2	n/a	n/a (alt)	7
Chung KP et al. [71]	2013	yes	66	E	ind	9–1–9	email	n/a	0.1	EV	n/a (alt)	8
Danner M et al. [72]	2011	yes	19 (12P, 7E)	E + P	g	9–1–9	f2f (workshop)	Expert Choice®	<0.1	EV, GGM	n/a	9
Diaz-Ledezma C et al. [107]	2014	yes	1	A	n/a	9–1–9	n/a	SuperDecisionsTM	0.1	n/a	yes (alt)	7
Diaz-Ledezma C, Parvizi J [73]	2013	yes	1	A	n/a	9–1–9	lit	SuperDecisionsTM	0.1	n/a	yes (alt)	8
Dolan JG et al. [25]	2013	yes	484	P	ind	9–1–9	f2f	Excel, Crystal Xcelsius, Expert Choice®	0.15	EV	n/a (alt)	9
Dou L et al. [61]	2015	yes	40	E	ind	1/9–1–9	delphi method	Expert Choice	0.1	n/a	n/a	8
Fang LF, Tung HH [104]	2010	yes	65	E	ind	n/a	questionnaire	SPSS	n/a	EV, GA	n/a	7
Guariguata L, Whiting D [110]	2011	yes	10	E	ind	5–1–5	questionnaire	n/a	n/a	n/a, GA	n/a (alt)	7
Hilgerink MP et al. [93]	2011	yes	7	E	ind + g	n/a	f2f (discussion)	Expert Choice®	n/a	EV, GGM	yes (alt)	8
Hou D et al. [67]	2014	yes	n/a	E	n/a	n/a	lit	n/a	0.1	n/a	n/a	4
Hsu HC et al. [90]	2010	yes	n/a	E	ind	5–1–5	f2f	MS Excel	n/a	EV, GGM	n/a (alt)	7
Hsu JC, Tang DH, Lu CY [63]	2015	yes	n/a	n/a	n/a	n/a	n/a	n/a	n/a	n/a	n/a	1
Hsu JC, Hsieh, C-Y, Yang Y-HK, Lu CY [65]	2015	yes	n/a	n/a	n/a	n/a	n/a	n/a	n/a	EV	n/a (alt)	2
Hu H et al. [68]	2010	yes	n/a	E	n/a	n/a	n/a	n/a	n/a	n/a, GGM	n/a	3
Hummel JM et al. [94]	2012	yes	6	E	ind	9–1–9	questionnaire	n/a	n/a	EV, GGM	n/a (alt)	7
Ijzerman MJ et al. [95]	2012	yes	86	E + P	ind + g	9–1–9	ppq	Expert Choice®	n/a	EV	n/a (alt)	8
Jaberidoost M et al. [66]	2015	yes	n/a	E	ind	1–9	questionnaire	Expert Choice®	n/a	EV, GGM	n/a	7
Joshi V et al. [74]	2011	yes	58	E	ind	1–11	online	n/a	0.1	n/a	n/a	7
Joshi V et al. [20]	2014	yes	422	E	ind	1–4	online	own software	0.1	n/a	n/a	8
Kadohira M [64]	2015	yes	313	E + C	ind	n/a	workshop, email	ASHtools.xls	0.15	n/a, GA	n/a (alt)	8
Karagiannidis A et al. [46]	2010	yes	n/a	E	g	1–9	n/a	Expert Choice®	0.1	EV	yes (alt)	8
Kitamura Y [47]	2010	yes	31	P	ind	1–7	online	n/a	0.3	EV	n/a (alt)	7
Krishnamoorthy K, Mahalingam M [100]	2015	yes	n/a	n/a	n/a	1/9–1–9	n/a	Expert Choice®	0.1	EV	yes (alt)	6
Kunasekaran V, Krishnamoorthy K [101]	2014	yes	n/a	n/a	n/a	1/9–1–9	n/a	Expert Choice®	0.1	n/a	yes (alt)	5
Kuruoglu E et al. [98]	2015	yes	96	P	ind	1–9	f2f	Expert Choice®	0.1	n/a, median of judgments	n/a	9
Lambooij MS, Hummel MJ [75]	2013	yes	66	E + P	ind	9–1–9	online	n/a	0.15 (in group)	EV, GA	n/a (alt)	8
Lee CW, Kwak NK [76]	2011	yes	n/a	E	n/a	n/a	n/a	n/a	0.1	EV	yes (alt)	5
Lee WC et al. [52]	2015	yes	200	C	n/a	1–9	n/a	Matlab	n/a	n/a	n/a (alt)	5
Li A-T, Lin J-W [77]	2014	yes	25	E	ind	1–9	email	Excel	0.1	n/a	n/a	8
Li C, Yu C [78]	2013	yes	n/a	E	n/a	n/a	n/a	n/a	n/a	n/a	yes (alt)	3
Lin RH, Chuang CL [91]	2010	yes	5	E	n/a	1–9	questionnaire	Expert Choice®	0.1	EV, GGM	n/a	8
Lu L et al. [53]	2015	yes	n/a	n/a	n/a	n/a	n/a	SPSS	n/a	n/a	yes	3
Maruthur NM et al. [111]	2015	yes	9	E	ind	“usual AHP scale”	computer	Expert Choice®	0.15	EV, GGM	yes (alt)	10
Mok H-P et al. [85]	2014	yes	n/a	E	n/a	n/a	n/a	n/a	0.01	n/a	n/a	3
Moslehi S, Atefi Manesh P, Sarabi Asiabar A [54]	2015	yes	5	E	n/a	1–9	n/a	K-Goepel Version 9.5.2012	0.072	n/a	n/a	6
Mühlbacher AC et al. [55]	2015	yes	1283	P	ind	9–1–9	online	n/a	0.006, 0.005	EV	n/a	8
Mühlbacher AC, Juhnke C, Kaczynski A [60]	2015	yes	24	P	ind + g	9–1–(−9)	group discussion	n/a	0.1	EV, consensus	n/a	8
Munoz DA, Nembhard HB, Kraschnewski Jennifer L [109]	2014	yes	1	A	ind	1–9	n/a	n/a	0.1	EV	n/a	7
Olivieri A et al. [79]	2012	yes	7	E	ind	1/9–1–9	questionnaire	n/a	n/a	n/a, GGM	n/a (alt)	7
Page K [80]	2012	yes	94	C	ind	9–1–9	ppq	SPSS	average at 0.3	EV	SD	10
Papadopoulos A et al. [56]	2015	yes	7	E	ind	1–9	n/a	n/a	0.1	EV, GGM	yes (alt)	8
Pecchia L et al. [81]	2011	yes	63	E	ind	5–1–5	online	n/a	0.2	EV, WM	n/a	8
Pecchia L et al. [26]	2013	yes	5	E	ind	5–1–5	ppq	n/a	0.1	EV	n/a	8
Perseghin P et al. [96]	2014	yes	11	E	g	1–9	email	n/a	n/a	n/a, GA	n/a	7
Petit J et al. [108]	2012	yes	n/a	A	n/a	9–1–9	n/a	n/a	0.1	EV	n/a (alt)	5
Ramezanpour B et al. [57]	2015	yes	24	E	g	1–9	n/a	n/a	0.1	EV	n/a	7
Reddy BP et al. [86]	2014	yes	8	E	ind + g	1/9–1–9	workshop, email	n/a	“standard”	EV, GGM and consensus	yes (alt)	9
Riepe MW [99]	2015	yes	42	E	ind	6–1–6	workshop	SPSS, spreadsheet file	0.1	n/a	n/a	8
Sharma PS et al. [82]	2011	yes	96	P	ind	9–1–9	f2f, (computer)	n/a	n/a	n/a	one-way for hybrid (alt)	7
Shojaei P et al. [87]	2014	yes	30	E	ind	9–1–1/9	f2f	Expert Choice®	0.1	EV, GGM	n/a (alt)	9
Smith J, Cook A, Packer C [48]	2010	yes	4 experienced horizon analysts	E	n/a	n/a	n/a	n/a	n/a	n/a	n/a (alt)	3
Šoltés V, Gavurová B [88]	2014	yes	16	E	ind	1–9	n/a	MS Excel	0.1 (for CI)	EV	n/a (alt)	8
Suner A et al. [83]	2012	yes	5	E	ind	9–1–9	online	Expert Choice®	0.1	EV	n/a	9
Taghipour H et al. [49]	2014	yes	40 hospitals	E	g	n/a	n/a	Expert Choice®, MS Excel	0.1	EV, WM	n/a (alt)	7
Tu C et al. [89]	2014	yes	41	E	ind	1–9	n/a	n/a	0.1	EV, GA	n/a (alt)	7
Uzoka FM et al. [97]	2011	yes	6	E	ind	9–1–9	n/a	n/a	0.2	EV, GA	n/a	7
Velmurugan R et al. [102]	2011	yes	n/a	n/a	n/a	9–1–9	n/a	n/a	0.1	AN	n/a (alt)	4
Wollmann D et al. [103]	2012	yes	400	C	ind	9–1–9	n/a	n/a	procedure by Silvac	n/a, GGM	n/a (alt)	7
Xu X, Cao Y, Luan X [58]	2014	yes	n/a	E	n/a	1–9	mobile phone app	n/a	n/a	n/a	n/a	4
Xu Y et al. [59]	2015	yes	954	P	ind	1–4	email	SAS	0.15	EV, arithmetic mean	n/a (alt)	9
Zhang S et al. [106]	2015	yes	n/a	E	n/a	1–5	n/a	JMP10.0	n/a	n/a	n/a	4
Zhu Q et al. [62]	2014	yes	9	E	ind	1–9	n/a	n/a	0.1	EV, GA	n/a	7

*P* patients, *C* potential consumers, *E* Experts, *n/a* not applicable, *ind* individual, *g* group, *online* online or web-based questionnaire, *f2f* face-to-face interview, *lit* literature, *quest* questionnaire (not further defined), *ppq* paper-pencil questionnaire, *email* mailed questionnaire, *CR* accepted consistency ratio, *EV* Eigenvector method, *GA* group average, *GGM* group geometric mean, *WM* weighted means, *AN* additive normalization method, *alt* alternatives included in the study, *SD* standard deviation

**Table 3:** List of all included studies

Author	Year	Title	Journal	Volume	Issue	Page
Ajami S, Ketabi S	2012	Performance evaluation of medical records departments by analytical hierarchy process (AHP) approach in the selected hospitals in Isfahan	Journal of Medical Systems	36	3	1165–1171
Angelucci E et al.	2008	Italian Society of Hematology practice guidelines for the management of iron overload in thalassemia major and related disorders	Hematology journal	93	5	741–752
Bahadori M et al.	2014	Assessing the service quality of Iran military hospitals: Joint Commission International standards and Analytic Hierarchy Process (AHP) technique	Journal of education and health promotion	3		98
Balestra G et al.	2007	AHP for the acquisition of biomedical instrumentation	Engineering in Medicine and Biology Society-Conference proceedings: 29th Annual International Conference of the IEEE			3581–3584
Barosi G et al.	2007	A unified definition of clinical resistance-intolerance to hydroxyurea in essential thrombocythemia: results of a consensus process by an intl. working group	Leukemia	21	2	277–280
Basoglu N, Daim TU, Topacan U	2012	Determining patient preferences for remote monitoring	Journal of Medical Systems	36	3	1389–1401
Baykasoğlu A, Dereli T, Yılankırkan N	2009	Application of cost-benefit analysis for surgical gown and drape selection: a case study	American Journal of Infection Control	37	3	215–226
Bi Y, Lai D, Yan H	2010	Synthetic evaluation of the effect of health promotion: impact of a UNICEF project in 40 poor western counties of China	Public Health	124	7	376–391
Brent A C et al.	2007	Application of the analytical hierarchy process to establish health care waste management systems that minimise infection risks in developing countries	European Journal of Operational Research	181		403–424
Cabrera-Barona P et al.	2015	A multi-criteria spatial deprivation index to support health inequality analyses	International journal of health geographics	14		11
Cancela J et al.	2015	Using the Analytic Hierarchy Process (AHP) to understand the most important factors to design and evaluate a telehealth system for Parkinson’s disease	BMC medical informatics and decision making	15	Suppl 3	S7
Carter KJ et al.	1999	Analysis of three decision-making methods: a breast cancer patient as a model	Medical Decision Making	19	1	49–57
Castro F et al.	1996	Sequential test selection in the analysis of abdominal pain	Medical Decision Making	16	2	178–183
Chang PY et al.	2006	Factors influencing medical students’ choice of specialty	Journal of the Formosan Medical Association = Taiwan yi zhi	105	6	489–496
Cheever MA et al.	2009	The prioritization of cancer antigens: a national cancer institute pilot project for the acceleration of translational research	Clinical Cancer Research	15	17	5323–5337
Chen L et al.	2014	Development of a decision support engine to assist patients with hospital selection	Journal of medical systems	38	6	59
Cho KT, Kim SM	2003	Selecting medical devices and materials for development in Korea: the analytic hierarchy process approach	International Journal of Health Planning and Management	18	2	161–174
Chung KP et al.	2013	Application of the analytic hierarchy process in the performance measurement of colorectal cancer care for the design of a pay-for-performance program in Taiwan	International Journal for Quality in Health Care	25	1	81–91
Cook DR, Staschak S, Green WT	1990	Equitable allocation of livers for orthotopic transplantation: an application of the Analytic Hierarchy Process	European Journal of Operational Research	48	1	49–56
Czaja S et al.	2003	A methodology for describing and decomposing complex psychosocial and behavioral interventions	Psychology and Aging	18	3	385–395
da Rocha LS, Sloane EB, Bassani JWM	2005	Optimal Medical Equipment Maintenance Service Proposal Decision Support System combining Activity Based Costing (ABC) and the Analytic Hierarchy Process (AHP)	Conference proceedings: 27th Annual International Conference of the IEEE Engineering in Medicine and Biology Society	7	7	103–106
Danner M et al.	2011	Integrating patients’ views into health technology assessment: AHP as a method to elict patient preferences	International Journal of Technology Assessment in Health Care	27	4	369–375
Dey P K, Hariharan S, Clegg B	2006	Measuring the operational performance of intensive care units using the analytic hierarchy process approach	International Journal of Operations&Production Management	26	8	849–865
Diaz-Ledezma C et al.	2014	Diagnosis of Periprosthetic Joint Infection in Medicare Patients: Multicriteria Decision Analysis	Clinical Orthopaedics and Related Research	URL: http://link.springer.com/article/10.1007%2Fs11999-014-3492-2 Accessed: 15 Feb 2014		
Diaz-Ledezma C et al.	2014	Diagnosis of Periprosthetic Joint Infection in Medicare Patients: Multicriteria Decision Analysis	Clinical Orthopaedics and Related Research	472	11	3275–3284
Diaz-Ledezma C, Parvizi J	2013	Surgical approaches for cam femoroacetabular impingement: the use of multicriteria decision analysis	Clinical Orthopaedics and Related Research	471	8	2509–2516
Dolan JG	2000	Involving patients in decisions regarding preventive health interventions using the analytic hierarchy process	Health Expectations	3	1	37–45
Dolan JG	1995	Are patients capable of using the analytic hierarchy process and willing to use it to help make clinical decisions	Medical Decision Making	15	1	76–80
Dolan JG	1990	Can decision analysis adequately represent clinical problems?	Journal of Clinical Epidemiology	43	3	277–284
Dolan JG	1989	Medical decision making using the analytic hierarchy process: choice of initial antimicrobial therapy for acute pyelonephritis	Medical Decision Making	9	1	51–56
Dolan JG et al.	2013	Patients’ Preferences and Priorities Regarding Colorectal Cancer Screening	Medical Decision Making	33	1	59–70
Dolan JG, Bordley DR	1994	Isoniazid prophylaxis-The Importance of Individual Values	Medical Decision Making	14	1	1–8
Dolan JG, Bordley DR	1993	Involving patients in complex decisions about their care: an approach using the analytic hierarchy process	Journal of General Internal Medicine	8	4	204–209
Dolan JG, Bordley DR, Miller H	1993	Diagnostic strategies in the management of acute upper gastrointestinal bleeding: patient and physician preferences	Journal of General Internal Medicine	8	10	525–529
Dolan JG, Frisina S	2002	Randomized controlled trial of a patient decision aid for colorectal cancer screening	Medical Decision Making	22	2	125–139
Dolan JG, Iadarola S	2008	Risk communication formats for low probability events--- an exploratory study of patient preferences	BMC medical informatics and decision making [electronic resource]	URL: http://www.ncbi.nlm.nih.gov/pmc/articles/PMC2330036/ Accessed 31 Dec 2013.		
Dolan JG, Isselhardt BJ, Cappuccio JD	1989	The analyic hierarchy process in medical decision making: a tutorial	Medical Decision Making	9	1	40-50
Dou L et al.	2015	An evaluation system for financial compensation in traditional Chinese medicine services	Complementary therapies in medicine	23	5	637–643
Eden KB et al.	2009	Patients were more consistent in randomized trial at prioritizing childbirth preferences using graphic-numeric than verbal formats	Journal of Clinical Epidemiology	62	4	415–424
Fang LF, Tung HH	2010	Comparison of nurse practitioner job core competency expectations of nurse managers, nurse practitioners, and physicians in Taiwan	Journal of the American Academy of Nurse Practitioners	22	8	409–416
Guariguata L, Whiting D	2011	The International Diabetes Federation diabetes atlas methodology for estimating global and national prevalence of diabetes in adults	Diabetes Research and Clinical Practice	94	3	322–332
Hannan EL, O’Donnell J, Freedland T	1981	A priority assignment model for standards and conditions in a long term care survey	Socio-economic Planning Sciences	15	6	277–289
Hariharan S et al.	2005	Application of analytic hierarchy process for measuring and comparing the global performance of intensive care units	Journal of Critical Care	20	2	117–125
Hariharan S et al.	2004	A new tool for measurement of process-based performance of multispecialty tertiary care hospitals	International Journal of Health Care Quality Assurance	17	6	302–312
Hilgerink MP et al.	2011	Assessment of the added value of the Twente Photoacoustic Mammoscope in breast cancer diagnosis	Medical Devices: Evidence and Research	4		107–115
Hou D et al.	2014	A real-time, dynamic early-warning model based on uncertainty analysis and risk assessment for sudden water pollution accidents	Environmental science and pollution research international	21	14	8878–8892
Hsu HC et al.	2010	Constructing area-level indicators of successful ageing in Taiwan	Health and Social Care in the Community	18	1	70–81
Hu H et al.	2010	Establishment and evaluation of a model of a community health service in an underdeveloped area of China	Public Health	124	4	206–217
Hummel JM et al.	2012	Predicting the Health Economic Performance of new non-fusion Surgery in Adolescent Idiopathic Scoliosis	Orthopaedic Research Society	30	9	1453–1458
Hummel JM et al.	2005	A multicriteria decision analysis of augmentative treatment of upper limbs in persons with tetraplegia	Journal of Rehabilitation Research & Development	42	5	635–544
Hummel JM et al.	2000	Medical technology assessment: the use of the analytic hierarchy process as a tool for multidisciplinary evaluation of medical devices	International Journal of Artificial Organs	23	11	782–787
Hsu JC et al.	2015	Net clinical benefit of oral anticoagulants: a multiple criteria decision analysis	PLoS One	10	4	e0124806
Hsu JC, Tang DH, Lu CY	2015	Risk-benefit assessment of oral phosphodiesterase type 5 inhibitors for treatment of erectile dysfunction: a multiple criteria decision analysis	International journal of clinical practice	69	4	436–443
Ijzerman MJ, van Til JA, Bridges JFP	2012	A Comparison of Analytic Hierarchy Process and Conjoint Analysis Methods in assessing treatment alternatives for stroke Rehabilitation	The Patient	5	1	45–56
Ijzerman MJ, van Til JA, Snoek GJ	2008	Comparison of two multi-criteria decision techniques for eliciting treatment preferences in people with neurological disorders	The Patient	1	4	265–272
Jaberidoost M et al.	2015	Pharmaceutical supply chain risk assessment in Iran using analytic hierarchy process (AHP) and simple additive weighting (SAW) methods	Journal of pharmaceutical policy and practice	8	1	9
Javalgi RG, Rao SR, Thomas EG	1991	Choosing a hospital: analysis of consumer tradeoffs	Journal of Health Care Marketing	11	1	12–22
Joshi V et al.	2011	Empirical investigation of radiologists’ priorities for PACS selection: an analytical hierarchy process approach	Journal of Digital Imaging	24	4	700–708
Joshi V et al.	2014	PACS Administrators’ and Radiologists’ Perspective on the Importance of Features for PACS Selection	Journal of digital imaging	27	4	486–495
Kadohira M et al.	2015	Stakeholder prioritization of zoonoses in Japan with analytic hierarchy process method	Epidemiology and infection	143	7	1477–1485
Karagiannidis A et al.	2010	A multi-criteria assessment of scenarios on thermal processing of infectious hospital wastes: a case study for Central Macedonia	Waste Management	30	2	251–262
Karamouz M et al.	2007	Developing a master plan for hospital solid waste management: a case study	Waste Management	27	5	626–638
Katsumura Y et al.	2008	Relationship between risk information on total colonoscopy and patient preferences for colorectal cancer screening options: Analysis using the Analytic Hierarchy Process	BMC Health Services Research	8	1	
Kitamura Y	2010	Decision-making process of patients with gynecological cancer regarding their cancer treatment choices using the analytic hierarchy process	Japan Journal of Nursing Science	7	2	148–157
Koch T, Ridgley M	1998	Distanced Perspectives: AIDS, Anencephaly, and AHP	Theoretical Medicine and Bioethics	19	1	47–58
Koch T, Rowell M	1999	The dream of consensus: finding common ground in a bioethical context	Theoretical Medicine and Bioethics	20	3	261–273
Krishnamoorthy K, Mahalingam M	2015	Selection of a suitable method for the preparation of polymeric nanoparticles: multi-criteria decision making approach	Advanced pharmaceutical bulletin	5	1	57–67
Kunasekaran V, Krishnamoorthy K	2014	Multi criteria decision making to select the best method for the preparation of solid lipid nanoparticles of rasagiline mesylate using analytic hierarchy process	Journal of advanced pharmaceutical technology & research	5	3	115–121
Kuruoglu E, Guldal D, Mevsim V, Gunvar T	2015	Kuruoglu, Emel; Guldal, Dilek; Mevsim, Vildan; Gunvar, Tolga	BMC medical informatics and decision making	15		63
Kwak NK, McCarthy K, Parker GE	1997	A human resource planning model for hospital/medical technologists: an analytic hierarchy process approach	Journal of Medical Systems	21	3	173–187
Lambooij MS, Hummel MJ	2013	Differentiating innovation priorities among stakeholder in hospital care	BMC Medical Informatics and Decision Making	URL: http://www.biomedcentral.com/1472-6947/13/91 Accessed 31 Dec 2013.		
Lee CW, Kwak NK	2011	Strategic Enterprise Resource Planning in a Health-Care System Using a Multicriteria Decision-Making Model	Journal of Medical Systems	35	2	265–275
Lee WC et al.	2015	A speedy cardiovascular diseases classifier using multiple criteria decision analysis	Sensors	15	1	1312–1320
Li A-T, Lin J-W	2014	Constructing core competency indicators for clinical teachers in Taiwan: a qualitative analysis and an analytic hierarchy process	BMC medical education	14		75
Li C, Yu C	2013	Performance Evaluation of Public Non-Profit Hospitals Using a BP Artificial Neural Network- The Case of Hubei Province in China	International Journal of Environmental Research and Public Health	10	8	3619–3633
Lin RH, Chuang CL	2010	A hybrid diagnosis model for determining the types of the liver disease	Computers in Biology and Medicine	40	7	665–670
Lu L et al.	2015	Assessment of regional human health risks from lead contamination in Yunnan province, southwestern China	PLoS One	10	3	e0119562
Maruthur NM et al.	2015	Use of the analytic hierarchy process for medication decision-making in type 2 diabetes	PLoS One	10	5	e0126625
Matsuda S	1996	An analysis of the Vietnamese system of occupational safety and health and setting priorities with the analytical hierarchy process	Occupational and Environmental Medicine	53	4	281–286
Matsuda S, Washino K	1998	How do the Japanese medical students evaluate the effectiveness of anti-smoking strategies- an application of the Analytic Hierarchy Process	Environmental Health and Preventive Medicine	3	2	73–77
Mok H-P et al.	2014	Development and validation of a convenient formula evaluating the value and applicability of medical literature in clinical practice	Pakistan journal of medical sciences	30	6	1377–1382
Moslehi S, Atefi Manesh P, Sarabi Asiabar A	2015	Quality measurement indicators for Iranian Health Centers	Medical journal of the Islamic Republic of Iran	29		177
Mühlbacher AC et al.	2015	Objective Criteria in the Medicinal Therapy for Type II Diabetes: An Analysis of the Patients’ Perspective with Analytic Hierarchy Process and Best-Worst Scaling	Gesundheitswesen			online
Mühlbacher AC, Juhnke C, Kaczynski A	2015	Patients’ Priorities in the Treatment of Neuroendocrine Tumours: An Analytical Hierarchy Process	Gesundheitswesen			online
Munoz DA, Nembhard HB, Kraschnewski JL	2014	Quantifying complexity in translational research: an integrated approach	International journal of health care quality assurance	27	8	760–776
Nuijten MJC, Kosa J	2004	Pricing of pharmaceuticals: Assessing the pricing potential by a pricing matrix model	The European Journal of Health Economics	5	2	110–115
Oddershede Herrera A, Carrasco González R, Barham Abu-Muhor E	2008	Multi-criteria Decision Model for Assessing Health Service Information Technology Network Support Using the Analytic Hierarchy Process	Computatión y sistemas	12	2	173–182
Olivieri A et al.	2012	Proposed definition of ‘poor mobilizer’ in lymphoma and multiple myeloma: an analytic hierarchy process by ad hoc working group Gruppo ItalianoTrapianto di Midollo	Bone Marrow Transplantation	47	3	342–351
Page K	2012	The four principles: can they be measured and do they predict ethical decision making	BMC Medical Ethics	13	10	online
Papadopoulos A et al.	2015	TDS exposure project: application of the analytic hierarchy process for the prioritization of substances to be analyzed in a total diet study	Food and chemical toxicology : an international journal published for the British Industrial Biological Research Association	76		46–53
Pecchia L et al.	2011	Analytic Hierarchy Process (AHP) for examining healthcare professionals’ assessments of risk factors	Methods of Information in Medicine	50	5	435–444
Pecchia L et al.	2013	User needs elicitation via analytic hierarchy process (AHP). A case study on a Computed Tomography (CT) scanner	BMC medical informatics and decision making [electronic resource]	13	2	
Perseghin P et al.	2014	A policy for the disposal of autologous hematopoietic progenitor cells: report from an Italian consensus panel	Transfusion	54	9	2353–2360
Petit J et al.	2012	Softening the Rule of Five- where to draw the line?	Bioorganic & Medicinal Chemistry	20	18	5343–5351
Ramezanpour B et al.	2015	Market implementation of the MVA platform for pre-pandemic and pandemic influenza vaccines: A quantitative key opinion leader analysis	Vaccine	33	35	4349–4358
Reddy BP et al.	2014	Prioritising public health guidance topics in the National Institute for Health and Care Excellence using the Analytic Hierarchy Process	Public Health	128	10	896–903
Richman MB et a.l	2005	A novel computer based expert decision making model for prostate cancer disease management	The Journal of Urology	174	6	2310–2318
Riepe MW	2015	Clinical preference for factors in treatment of geriatric depression	Neuropsychiatric disease and treatment	11		25–31
Ross ME, Nydick RL	1992	Selection of licensing candidates in the pharmaceutical industry: an application of the analytic hierarchy process	Journal of Health Care Marketing	12	2	60–65
Sharma PS et al.	2011	Subjective risk vs. objective risk can lead to different post-cesarean birth decisions based on multiattribute modeling	Journal of Clinical Epidemiology	64	1	67–78
Shin T et al.	2009	The comparative evaluation of expanded national immunization policies in Korea using an analytic hierarchy process	Vaccine	27	5	792–802
Shojaei P et al.	2014	Ranking the effects of urban development projects on social determinants of health: health impact assessment	Global journal of health science	6	5	183–195
Singh S, Dolan JG, Centor RM	2006	Optimal management of adults with pharyngitis-a multi-criteria decision analysis	BMC Medical Informatics and Decision Making	6	14	online
Smith J, Cook A, Packer C	2010	Evaluation criteria to assess the value of identification sources for horizon scanning	International Journal of Technology Assessment in Health Care	26	3	348–353
Šoltés V, Gavurová B	2014	The functionality comparison of the health care systems by the analytical hierarchy process method	E + M Ekonomie a Management	17	3	100–117
Suner A et al.	2012	Sequential decision tree using the analytic hierarchy process for decision support in rectal cancer	Artificial Intelligence in Medicine	56	1	59–68
Taghipour H et al.	2014	On-site or off-site treatment of medical waste: a challenge	Journal of environmental health science & engineering	12	1	68
Tan X et al.	2007	Evaluation of the Effect of a Health Education Campaign of HIV by Using an Analytical Hierarchy Process Method	International journal of Environmental Research and Public Health	4	3	254–259
Tarimcilar MM, Khaksari SZ	1991	Capital budgeting in hospital management using the analytic hierarchy process	Socio-economic Planning Sciences	25	1	27–34
Tu C et al.	2014	Application of the analytic hierarchy process to a risk assessment of emerging infectious diseases in Shaoxing city in southern China	Japanese journal of infectious diseases	67	6	417–422
Tzung TY et al.	2007	Decision factors and the recognition of medical specialty in patients receiving cosmetic laser and intense pulsed light treatment	Dermatologic Surgery	33	12	1488–1493
Uzoka FM et al.	2011	An experimental comparison of fuzzy logic and analytic hierarchy process for medical decision support systems	Computer Methods and Programs in Biomedicine	103	1	10–27
van Til JA et al.	2008	The use of the analytic hierarchy process to aid decision making in acquired equinovarus deformity	Archives of Physical Medicine and Rehabilitation	89	3	457–462
Velmurugan R, Selvamuthukumar S, Manavalan R	2011	Multi criteria decision making to select the suitable method for the preparation of nanoparticles using an analytical hierarchy process	Die Pharmazie	66	11	836–842
Wang KI et al.	2007	Analysis of senior medical students’ preferences in specialty choice a survey in a medical school in northern Taiwan	Chang Gung Medical Journal	30	4	339–353
Weingarten MS et al.	1997	A pilot study of the use of analytic hierarchy process for the Selection of Surgery Residents	Academic medicine: journal of the Association of American Medical Colleges	72	5	400–402
Wollmann D et al.	2012	Evaluation of health service providers by consumers through the Analytic Hierarchy Process Method	Revista de Saúde Pública	46	5	777–783
Wu C, Lin C, Chen H.	2007	Optimal selection of location for Taiwanese hospitals to ensure a competitive advantage by using the analytic hierarchy process and sensitivity analysis	Building and Environment	42		1431–1444
Xu X, Cao Y, Luan X	2014	Application of 4G wireless network-based system for remote diagnosis and nursing of stomal complications	International journal of clinical and experimental medicine	7	11	4554–4561
Xu Y et al.	2015	Comparison of patient preferences for fecal immunochemical test or colonoscopy using the analytic hierarchy process	BMC health services research	15		175
Zhang S et al.	2015	Indicators for Environment Health Risk Assessment in the Jiangsu Province of China	International journal of environmental research and public health	12	9	11012–11024
Zhu Q et al.	2014	The spatial distribution of health vulnerability to heat waves in Guangdong Province, China	Global Health Action	7		25051

## Abbreviations

AHP: Analytic Hierarchy Process
CHERH: Center for Health Economics Research Hannover
CONSORT: Consolidated Standards of Reporting Trials
CR: Consistency Ratio
EV: Eigenvector method
IQWiG: Institute for Quality and Efficiency in Health Care
ISPOR: International Society for Pharmacoeconomics and Outcomes Research
PRISMA: Preferred Reporting Items for Systematic Reviews and Meta-Analyses
